# Effect of Solidification and Hot Rolling Processes on Wear Performance of TiC-Reinforced Wear-Resistant Steel

**DOI:** 10.3390/ma15030729

**Published:** 2022-01-19

**Authors:** Xiangtao Deng, Qi Wang, Long Huang, Yi Cao, Zhaodong Wang

**Affiliations:** 1 State Key Laboratory of Rolling and Automation, Northeastern University, Shenyang 110819, China; huanglong420@163.com (L.H.); zhdwang@mail.neu.edu.cn (Z.W.); 2 Liaoning Key Laboratory of Optimized Utilization for Non-Associated Lean Iron Ore, Liaoning Institute of Science and Technology, Benxi 117004, China; neu13358878516@163.com

**Keywords:** TiC particle, wear resistant steel, solidification rate, rolling compression ratio, wear mechanism

## Abstract

As a commonly reinforcing phase in wear-resistant materials, TiC is often added into wear-resistant materials to improve the wear resistance. The independently developed stepped molds with variable thicknesses were used to prepare the TiC-reinforced steels with the same composition though melt solidification processing to study the effect of the solidification rate on the particle size and wear performance. The effect of the hot rolling compression ratio on the particle size and wear performance was also studied. The length and aspect ratios of the particles in heat-treated TiC-reinforced steels with different billet thicknesses and rolling compression ratios were measured. With the increasing in the billet thickness and the decreasing in the rolling compression ratio, the length and aspect ratio of the particles increased in heat-treated TiC-reinforced steels, and the hardness decreased slightly. The three-body abrasive wear behavior of the TiC-reinforced steels was conducted using a standard dry sand rubber wheel wear testing procedure, and the modeling of the wear mechanism was established. The particle size is the main factor affecting wear resistance when the hardness of TiC-reinforced steels is similar. When the particles size is moderate, about 2–6 μm, the particle can break the sand tip and hinder the sand tip from sliding on the surface. In this manner, the mass loss decreased and the wear resistance improved. The large particles will be broken easily by the abrasive, and the small particles are removed easily by the abrasive in the wear process. So, the large and small particles cannot effectively prevent the damage of the abrasive to the matrix, and they have less of an effect on improving wear resistance.

## 1. Introduction

Adding hard particles such as carbide [1], nitride, boride [2], or oxide [3] into wear-resistant materials can improve the wear resistance of the material without increasing the hardness. TiC is especially suitable for steel due to its high hardness (2859–3200 HV) [4], low density (≈4.93 g/cm^3^), high melting point (≈3430 K), and high wear resistance [5,6]. The two main production methods of particle-reinforced steels could be classified into solid state (powder metallurgy [7], self-propagation high-temperature synthesis [8], mechanical alloying [9], and carbon-thermal reduction [10]) and molten/casting state methods (adding TiC to Fe-C [11,12], adding ferrotitanium to molten Fe-C [13,14], adding C to Fe-Ti [15], and adding Ti to molten Fe-C [16]). Furthermore, the TiC coating with high wear resistance can be obtained by laser cladding [17,18] or welding [19,20] on the surface of the material to achieve the purpose of protecting the material. Thereinto, the advantage of the solid state and laser cladding or welding method is that the materials with high-volume fraction TiC can be prepared, but the disadvantages of the solid-state method are that the process is complex, the uniformity of TiC is poor, and it will have many defects. The main disadvantage of the laser cladding or welding method is that the thickness of the TiC coating is thin. To meet the large-scale industrial production in the particle-reinforced steels, the molten/casting state method is the best process.

The effect of the size, the volume fraction, the Ti/C ratio, and the distribution of particles on the wear resistance has attracted a lot of research interest [21]. In our previous study [22], TiC-reinforced low alloy wear-resistant steel was developed, which has better wear resistance than traditional low alloy wear-resistant steel, and the wear resistance of TiC-reinforced steels increased with the increase in reinforcing phase volume fraction [23]. Additionally, the evolution of TiC formed during solidification at different solidification rates by a confocal laser scanning microscope and the sliding wear behavior of TiC-reinforced steels were investigated [24]. Du et al. [25] studied the effect of solidification rate on micro-TiC precipitation by different casting techniques, but the effect of solidification rate on wear resistance was not considered. With the increase in the rolling compression ratio (H_slab_/H_plate_, where the H_slab_ was the thickness of the slab, and H_plate_ was the thickness of the plate), the large boride was broken into refined particles, and the ductility and toughness of boride-reinforced austenitic stainless steel were significantly improved [26]. The solid-state method can well achieve the regulation of particle size. Particle size can be controlled by controlling the particle size of the powder [27] and the temperature and time of hot isostatic pressing [28], and combustion synthesis can also be controlled by controlling the particle size of the powder [29]. However, considering the need for industrial large-scale production, the liquid-state method has incomparable advantages compared with the solid-state method. However, to the best of our knowledge, the effect of billet solidification rate and rolling compression ratio on the wear resistance of particle-reinforced steels has received little attention.

Continuous casting is generally used to obtain billets in industrial production, so the solidification rate can be controlled by controlling the thickness of billets and water cooling conditions. In this paper, the solidification rate was controlled by the independently developed stepped molds with variable thickness, and the effect of the solidification rate on the particles of the billets, and the particles, microstructure, and wear properties after hot rolling and heat treatment was studied. At the same time, in industrial production, the same thickness of the casting billet is used through different rolling processes to obtain different plate thicknesses, so it is necessary to study the effect of compression ratio on the particles, microstructure, and wear properties after hot rolling and heat treatment. The research of this paper can provide a certain theoretical basis for the regulation of micron TiC particles in industrial production.

## 2. Materials Preparation and Experimental Procedure

### 2.1. Materials Production

The used TiC-reinforced steels with chemical composition 0.3 C, 0.5 Mn, 0.6 Ti, 0.3 Mo, 0.25 Si, 0.0015 B, bal. Fe, in wt % were produced in a vacuum induction furnace (VIM) and then cast in an independently developed stepped molds with variable thickness. One constant-thickness billet with a thickness, width, and length of 90, 120, and 640 mm, and two stepped billets with variable thicknesses of 10 + 60 mm and 5 + 30 mm were prepared. The widths and lengths of the two stepped billets are 120 and 640 mm, respectively, and the position of the thickness change is in the middle of the length. The three billets had the same chemical composition and length.

**Figure 1:**
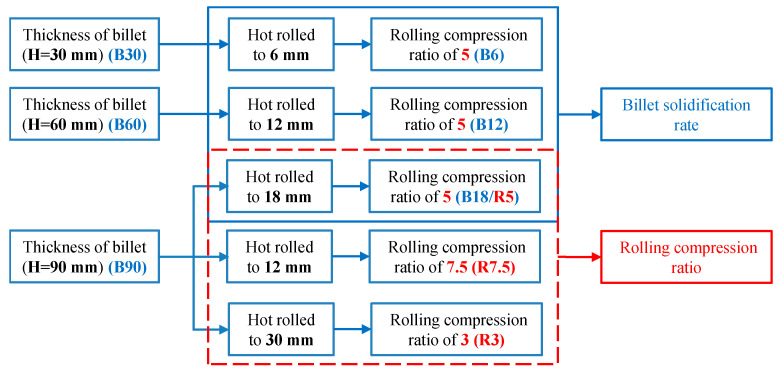
The sample identification (ID) and the corresponding processing routes of the TiC-reinforced steels.

The solidification rates decreased with the increase in billet thickness, which affected the microstructure and TiC particles formed in the TiC-reinforced steels. The TiC-reinforced steels were homogenized at 1200 °C for 3 h and then hot rolled by two-stage controlled rolling [30] with two reverse rolling mills and cooled by air to ambient temperature. The 30 mm-thick, 60 mm-thick, and 90 mm-thick billets (sample ID: B30, B60, B90) were hot rolled with a rolling compression ratio of 5 (sample ID: B6, B12, B18) to study the effects of the billet solidification rate on the particles and wear resistance of the TiC-reinforced steels. Three 90 mm-thick billets were hot rolled under different rolling compression ratios of 7.5, 5, and 3 (sample ID: R7.5, R5, R3) to study the effects of the rolling compression ratio on the particles and wear resistance of the TiC-reinforced steels, and the sample ID and the corresponding processing routes are summarized in Figure 1. The TiC-reinforced steels after hot rolling were heated to 900 °C to austenization in a box-type resistance furnace and followed by water quenching to room temperature; then, they were tempered at 200 °C.

### 2.2. Experimental Procedures

In order to observe the morphology of TiC particles clearly, the morphology of the particles was obtained though non-erosion specimens, and the microstructure was studied though erosion specimens by using a Zeiss Ultra-55 scanning electron microscopy (SEM) under an acceleration voltage of 15 kV. The etching solution was a solution of nitric acid (4%) and alcohol solution (96%). The size of the particles was measured using the software Image-Pro Plus (IPP; ten SEM photos were taken into account for the statistics of each sample; covered at least 1300 particles). Macro-Vickers hardness measurements were conducted under a load of 30 kg by using a KB3000BVRZ-SA hardness tester. Ten individual measurements were performed, and the average value was provided.

The abrasive wear samples with a thickness, width, and length of 7, 25.5, and 75 mm were cut from heat-treated TiC-reinforced steels, and the length direction is along the rolling direction. The experiment was conducted under ambient temperature by using a dry sand/rubber wheel wear testing machine (MLG-130B). The experiment was carried out in accordance with the ASTM standard test method G65 Procedure B [31]. The experiment parameters were as follows: the load of 130 N, the rotational speed of 200 rpm, the cycle number of 2000 r, the rubber wheel diameter of 228.6 mm, the rubber wheel hardness of 60 Shore A, quartz sand with sizes in the range of 212–300 µm, and the sand flow rate of 300 g/min. Three parallel experiments were taken for different steels, and the average value was provided. Before and after the tests, all experimental samples were cleaned to wipe off the attached abrasive and impurities by using an ultrasonic cleaner in alcohol for 15 min. The weight of wear samples after cleaning was measured by a SECURA225D-1CN electronic balance. The wear mechanism was analyzed by observing the wear morphology with SEM.

## 3. Results and Discussions

**Figure 2:**
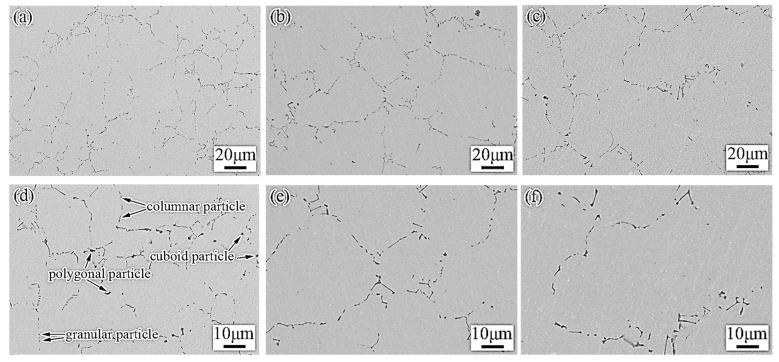
Morphologies of particles in the billets with different thicknesses: (**a**,**d**) B30; (**b**,**e**) B60; (**c**,**f**) B90.

Figure 2 shows the morphologies and distribution of particles in the billets with different thicknesses. The TiC particles have different morphologies including majority columnar, polygonal, granular, and cuboid shapes. The particles show a nonuniform distribution in the matrix, because the TiC particles are attributed to eutectic reaction. With the increasing of the billet thickness, the solidification rate decreased, the secondary dendrite arm spacing (SDAS) increased, and dendritic coarsening occurred [26,30]. Image analysis was taken of the particle morphology obtained by SEM to obtain Feret diameters [32]. Springer et al. used area to describe the irregular TiB2 particle [33,34], but area cannot accurately reflect the morphology of particles with different shapes. For comparison, Rana [35] et al. reported a detailed analysis of particle characteristics, consisting of diameter, length, aspect ratio, area, number density, and volume fraction. Since the chemical composition of TiC-reinforced steels were the same, it can be regarded that the volume fraction of particles was also similar. Therefore, the number density of particles was related to the size of the particles. By comparing the statistical methods of particles in different reference literatures, the aspect ratio and the average length of particle were provided to describe the particle characteristics. The effect of improving wear resistance was small when the particle size was in the submicron range [34], so only the particles longer than 1 μm were counted in this paper. The Feret diameters were measured (passing through the center of the particle, the diameter in any direction is called a Feret diameter); the maximum value was taken as the length of the particle, and the minimum value was taken as the width of the particle, and the ratio of the length and width was taken as the aspect ratio.

**Figure 3:**
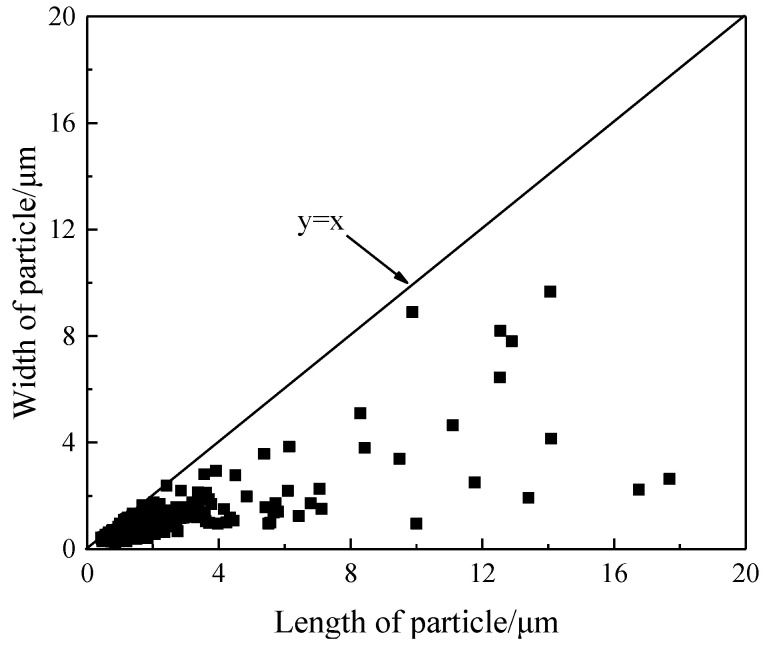
Scatter diagram of particles’ length and width in the billet with thickness of 30 mm.

Within the scatter diagram of the particles’ length and width (Figure 3), the aspect ratio of the particles near the line (y=x) is equal to 1, and any deviation away from 1 indicates a deviation from circularity, the departure degree increases as long as the aspect ratio increases; in this case, the particles are basically columnar in shape. The average length of the TiC particles in the different thickness billets was within 2.20–3.58 μm and increased with the billet thickness increasing. Meanwhile, the volume fraction and the average aspect ratio of the particles have little change at approximately 1.20% and 2.6, respectively.

### 3.1. Effect of Solidification Rate on the Wear Performance

**Figure 4:**
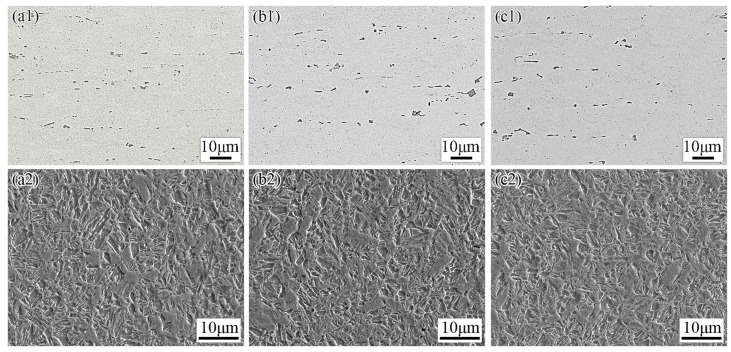
Morphologies of particles (**a1**–**c1**) and microstructure (**a2**–**c2**) in heat-treated TiC-reinforced steels with different billet thickness: (**a**) B6; (**b**) B12; (**c**) B18.

The morphologies of particles in the heat-treated TiC-reinforced steels with different billet thickness were observed by SEM, and the morphologies of the particles and microstructure are shown in Figure 4. After hot rolling, the particle size decreased, and the distribution is more uniform compared with the particles in the billet. The structure of heat-treated TiC-reinforced steel is lath martensite with many particles.

**Figure 5:**
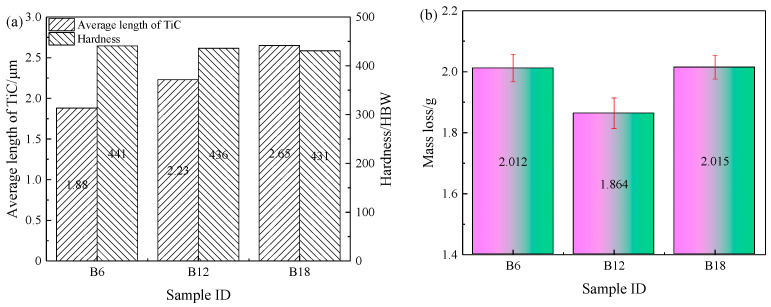
Image analysis result, hardness and wear results of heat-treated TiC-reinforced steels with different billet thicknesses: (**a**) image analysis result and hardness; (**b**) wear results.

Figure 5 shows the image analysis results of the particles as well as the hardness and wear results of heat-treated TiC-reinforced steels with different billet thicknesses. The average length of particles after hot rolling was relatively smaller by approximately 15% than that in the billets. The average length of particles increased with the billet thickness increasing under the same rolling compression ratio, but the average aspect ratios of the particles were similar: about 2.4. The volume fraction of TiC decreased more lightly than that in the billet, because the submicron smaller particles increased after hot rolling. The image analysis results of particles showed that the change trend was consistent with billets. When the billet thickness increases, the hardness of heat-treated TiC-reinforced steels with different billet thicknesses changed slightly: from 441 to 431 HBW. The wear mass loss of heat-treated TiC-reinforced steels with different billet thickness is indicated in Figure 5b; three parallel experiments were taken for different steels, and the average value was provided. The experimental result was stable, and the standard deviation of mass loss was ±0.05 g. The wear mass loss of the heat-treated B12 steel was the minimum, but the hardness was not the minimum. This result was inconsistent with most research results, and it is generally believed that the wear resistance increased with the hardness increasing [36,37], which indicated that there were other factors that affect the wear resistance.

**Figure 6:**
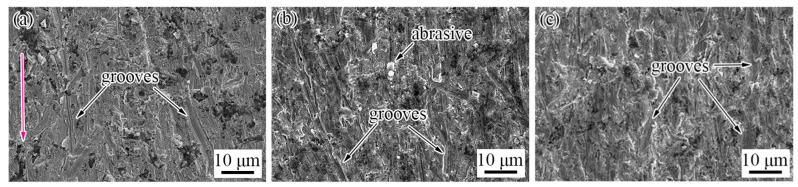
Wear morphology of heat-treated TiC-reinforced steels with different billet thickness: (**a**) B6; (**b**) B12; (**c**) B18. The red arrow indicates wear direction.

The wear morphology of TiC-reinforced steels was conducted using SEM (Figure 6), which reveals that the micro-cutting, furrow, and strain fatigue effects were the main wear mechanisms. More grooves that were caused by the micro-cutting mechanism can be found in the wear morphology of the heat-treated B6 steel (Figure 6a). The materials’ removal requires only one pass of groove caused by the micro-cutting mechanism, which causes more serious mass loss; thus, the wear resistance of the heat-treated B6 steel was poor. The worn surface features more furrow wear mechanism characteristics of the heat-treated B12 steel (Figure 6b); meanwhile, the abrasive tip was also broken and embedded in the matrix, which prevented the grooves from extending and reduced the mass loss of the materials. The worn surface shows more micro-cutting mechanism wear characteristics of the heat-treated B18 steel (Figure 6c). These results explain the relatively good wear resistance of the heat-treated B12 steel.

### 3.2. Effect of Rolling Compression Ratio on the Wear Performance

**Figure 7:**
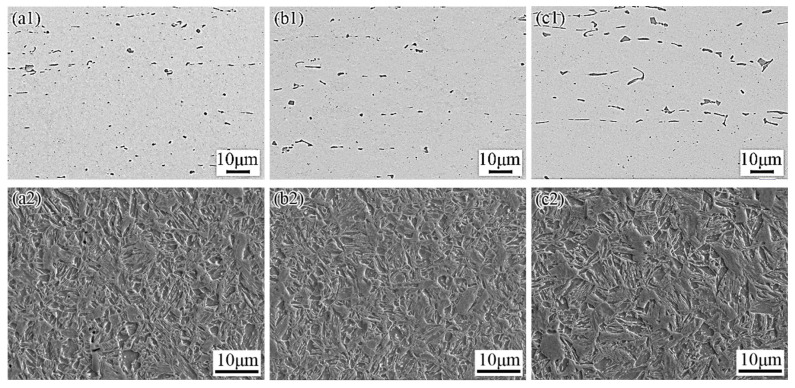
Morphologies of particles (**a1**–**c1**) and microstructure (**a2**–**c2**) of in heat-treated TiC-reinforced steels with different rolling compression ratios: (**a**) R7.5; (**b**) R5; (**c**) R3.

**Figure 8:**
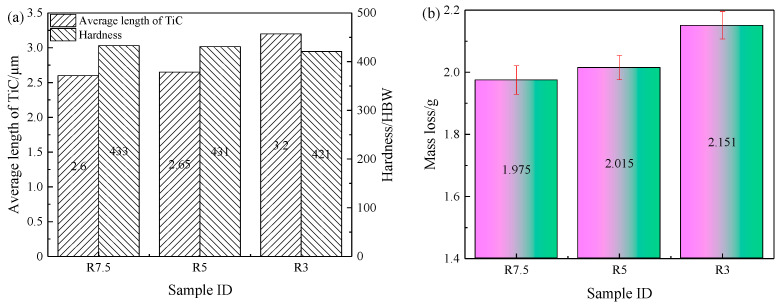
Image analysis result, hardness, and wear results of heat-treated TiC-reinforced steels with different rolling compression ratios: (**a**) image analysis result and hardness; (**b**) wear results.

The morphologies of particles in heat-treated TiC-reinforced steels with different rolling compression ratios are shown in Figure 7. A slight aggregation with particles was found for the heat-treated R3 steel, and increasing the rolling compression ratio made the distribution of particles more uniform. The aspect ratio and average length of particles in the TiC-reinforced steels decreased as the rolling compression ratio increased. However, the average length of particles in the heat-treated R5 steel was observed to be similar with that in the heat-treated R7.5 steel, and the average length of particles in the heat-treated R3 steel was 1.2 times than those of the former two. The detailed analysis of particles data (Figure 5a and Figure 8a) revealed that the volume fraction and average aspect ratio of particles were similar in all TiC-reinforced steels; hence, the average length of particles was the primary factor influencing the wear resistance in TiC-reinforced steels.

After heat treatment, the microstructure of the heat-treated TiC-reinforced steels is tempered martensite, as shown in Figure 7. The size of original austenite grain decreased with the rolling compression ratio increasing, and the hardness of the heat-treated TiC-reinforced steels increased slightly. However, the hardness of the heat-treated R5 steel was observed to be similar with that of the heat-treated R7.5 steel. The wear resistance increased with the hardness increasing (Figure 8b), which was consistent with most research results, but the hardness of experimental steels decreased slightly, so the change had no significant influence on the wear resistance.

**Figure 9:**
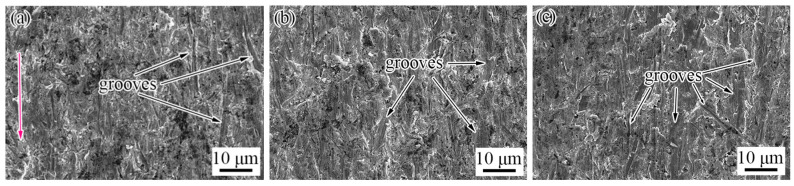
Wear morphology of heat-treated TiC-reinforced steels with different rolling compression ratios: (**a**) R7.5; (**b**) R5; (**c**) R3. The red arrow indicates the wear direction.

The worn surfaces of the heat-treated R5 and R7.5 steels (Figure 9a,b) present more micro-cutting mechanism wear characteristics. Figure 9c shows that the grooves were the most and the widest of the heat-treated R3 steel, so the wear morphology revealed that the wear resistance of the heat-treated R3 steel was the worst. The maximum width of the groove was 6 μm in Figure 9c, which was more than the average length of particles; therefore, both the matrix and particles were removed by grooves. The width of most of the grooves was below 2 μm, so the large particles can hinder the sand tips from sliding on the surface, and the small particles have less of an effect on improving the wear resistance.

### 3.3. Discussion

**Figure 10:**
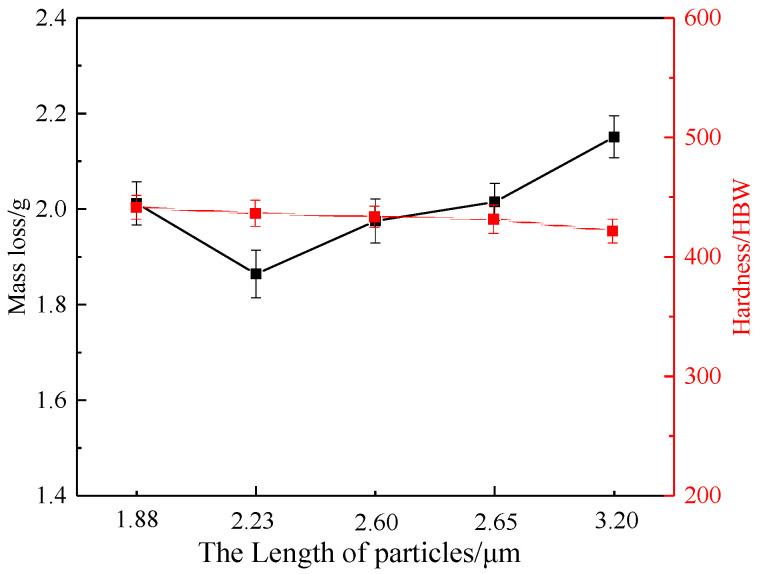
The relationship between the average length of particles, hardness, and mass loss of the TiC-reinforced steels.

Based on the above analysis, the volume fraction of TiC was similar in all TiC-reinforced steels. The change trend of the average aspect ratio of TiC was consistent with the average length of particles, but the average aspect ratio changed slightly. Thus, the average length of particles and hardness were the main influencing factors in wear resistance. To discuss the factors that affect the wear resistance, the relationship between the average length of particles, hardness, and mass loss of heat-treated TiC-reinforced steels is shown in Figure 10. The wear resistance first increased and then decreased with the hardness decreasing, which was inconsistent with most of the research results. The wear resistance of the TiC-reinforced steel with 436 HBW was 1.15 times that of the TiC-reinforced steel with 421 HBW. However, in our previous study [37], the wear resistance of the TiC-reinforced steel with 436 HBW was 1.05 times that of the TiC-reinforced steel with 421 HBW when the size of particles is similar, so the particle size played an important role in wear resistance.

**Figure 11:**
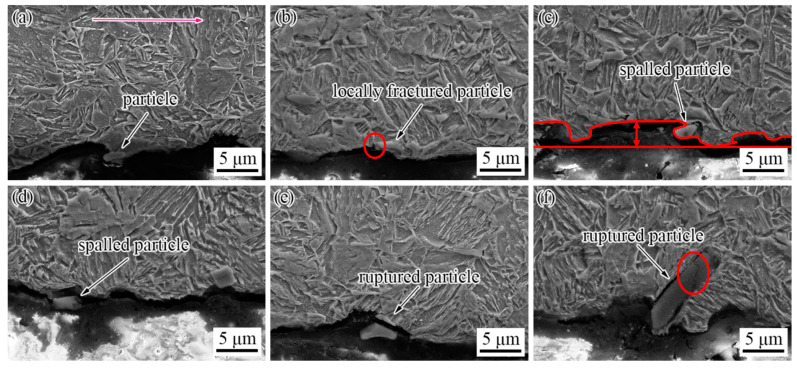
SEM micrographs of longitudinal sections of the TiC-reinforced steels: (**a**) complete particle; (**b**) locally fractured particle; (**c**,**d**) spalled particle; (**e**,**f**) ruptured particle. The red arrow indicates the wear direction.

To analysis the influence of the particle size on wear, the micrographs of longitudinal sections of the TiC-reinforced steels were performed by SEM. The longitudinal section sample was obtained by electro-discharge machining from the wear sample along the wear direction, and the particle states on the wear area were observed. As indicated in Figure 11, the particles standing out on the worn surface have different states including locally fractured particles, ruptured particles, and spalled particles. Figure 11 confirms that the depth of the groove (the distance of the two red lines in Figure 11c) becomes shallow when the abrasive tip glides through the particle, because the particles’ hardness (2859–3200 HV) is larger than the abrasive’s hardness (1100~1300 HV); thus, the particles can reduce the damage of the abrasive to the specimen.

Although the particles’ hardness is much larger than the abrasive hardness, the particles will be destroyed after repeated impacting by the abrasive. As shown in Figure 11b, the particle has been locally broken, and a small part has fallen off. When the surrounding matrix was insufficient to support the particles, the particles will be peeled off from the matrix, as shown in Figure 11c,d. When the interfacial bonding strength of the matrix and particle was strong, the particle will be ruptured (Figure 11e) rather than peeled off from the matrix, and a small part was still bonded with the matrix. This was due to the Ti-containing particle being a hard brittle phase with irregular shape, and the stress concentration between particle and surrounding matrix was caused by their different thermal expansion coefficients [38]. The thermal expansion coefficient of steel materials is generally 12 × 10^−6^/°C, while that of TiC is only 7 × 10^−6^/°C, so the volumetric shrinkage of TiC particles during the cooling process is much smaller than that of the matrix, which will form compressive stresses. The larger the particle size, the greater the stress between the particles and the surrounding matrix. The Griffith–Orowan formula indicates that the larger the particle size, the easier the crack initiation and propagation at the interface between the particles and the matrix. At the same time, a large number of tangled dislocations and a small amount of micro-crack extensions from inside the larger particles to the matrix can also be observed [39]. When the particle size was large, about 10 μm (Figure 11f), the resistance to crack propagation through the particle–matrix interface was decreased; thus, the interface crack can be observed between the particles and matrix, and the particle will be ruptured more easily. Interestingly, the size of all the particles in longitudinal sections was more than 2.5 μm, which indicated that the large particles are more favorable for improving the wear resistance. The wear morphology also proves this point. The maximum width of the groove was 6 μm and the width of most of the grooves was about 2 μm wide, so particles smaller than 2 μm were easily removed by the abrasive. According to the above analysis, medium-sized (2~6 μm) particles are more beneficial for improving wear resistance. The particle size of the best wear resistance can be obtained under the experimental conditions in this paper, which is similar to that previously reported [28,29]. However, this paper excludes the influence of material composition and hardness on wear resistance, and the main factor affecting the wear resistance in the wear test is the particle size; thus, the obtained results are more reliable. At the same time, a very thin deformation layer can be observed, and the thickness of the deformation layer was within 1 μm. This is because in the abrasive wear process, the wear mechanism was mainly furrow and strain fatigue effects with different degrees of micro-cutting. Furrow and micro-cutting mechanisms continuously removed materials, so it was difficult for the deformation layer to be retained.

**Figure 12:**
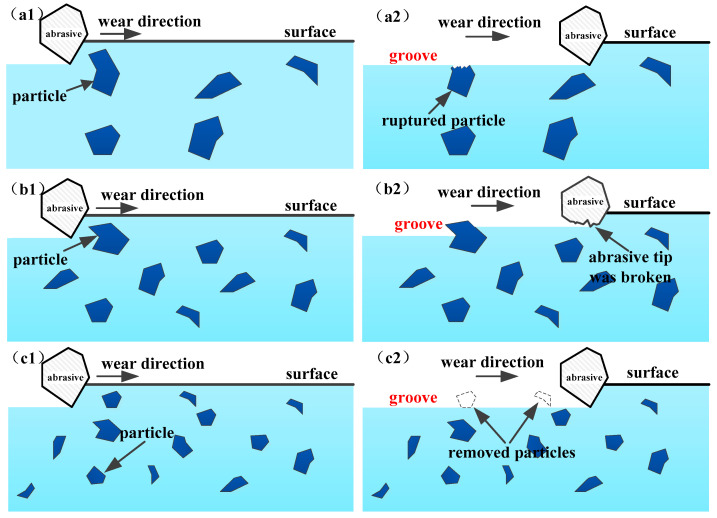
The relationship between particle size and wear mechanism of experiment steels: (**a1**,**a2**) when the particle size is large; (**b1**,**b2**) when the particle size is moderate; (**c1**,**c2**) when the particle size is small.

A schematic drawing (Figure 12) illustrates the effect of particle size on the wear mechanism. The large particles will be broken easily by the abrasive (Figure 12a), so large particles cannot effectively prevent the damage of the abrasive to the matrix, and the wear resistance decreases. The hardness of the particle is much larger than that of the abrasive, so the moderately sized (2–6 μm) particles can break the abrasive tip and prevent the groove from extending, as shown in Figure 6b and Figure 12b. The small particles are removed by the abrasive in the wear process, as shown in Figure 12c. The fine particles cannot play a role in preventing the abrasive from sliding, which has less of an effect on improving the wear resistance.

## 4. Conclusions

(1) The solidification rate and particle size increased with the thickness of the billet, and the average length of particles was 2.20–3.58 μm in the 30–90 mm-thick billets; meanwhile, the average aspect ratio and the volume fraction of the particles were similar.
(2) The average length, average aspect ratio, and the volume fraction of the particles after hot rolling increased with the increasing of the billet thickness, and they decreased with the increasing of the rolling compression ratio. However, the average aspect ratio and the volume fraction of the particles in all the billets after hot rolling were similar.
(3) The wear mechanisms of TiC-reinforced steels under the conditions of dry sand/rubber wheel abrasive wear were micro-cutting, furrow, and strain fatigue effects. The wear resistance first increased and then decreased with the decreasing of the hardness, which violated most of the research results; thus, the effect of the particle size on the wear resistance must be considered.
(4) The small particles were easily removed by the abrasive, and the large particles were broken easily by the abrasive; thus, they have a less of an effect on improving the wear resistance. The moderately sized (2–6 μm) particles were shown more effective at breaking the abrasive tip and preventing the groove from extending.

## Data Availability

Not applicable.
